# A Polymer Nanocomposite with Strong Full‐Spectrum Solar Absorption and Infrared Emission for All‐Day Thermal Energy Management and Conversion

**DOI:** 10.1002/advs.202308200

**Published:** 2024-02-11

**Authors:** Xiangxin Li, Zipeng Zhang, Xueting Zhang, Yanxia Cao, Yanyu Yang, Wanjie Wang, Jianfeng Wang

**Affiliations:** ^1^ School of Materials Science and Engineering Zhengzhou University Zhengzhou 450001 China

**Keywords:** full‐spectrum absorption, heating and cooling, melt blending, polymer/MXene nanocomposites, uninterrupted thermoelectric conversions

## Abstract

Realizing efficient energy utilization from the heat source of the sun and the cold source of outer space is of great significance for addressing the global energy and environmental crisis. Materials with ideal full‐spectrum solar absorption and infrared emission are highly desirable for adapting to the continuous weather dynamic throughout the day, nonetheless, their development remains challenging. Here, a polymer nanocomposite with full‐spectrum strong solar (280–2500 nm) absorption ranging from 88.8% to 94.8% with an average value of 93.2% and full‐spectrum high infrared (8–13 µm) emission ranging from 81.3% to 90.0% with an average value of 84.2%, is reported by melt‐processing polypropylene and uniformly dispersed low‐loading MXene nanosheets (1.9 vol%). The nanocomposite can achieve daytime photothermal enhancement of ≈50 °C and nighttime radiative cooling of 8 °C. The temperature difference throughout the day ensures all‐day uninterrupted thermoelectric generation, yielding a power density output of 1.5 W m^−2^ (daytime) and 7.9 mW m^−2^ (nighttime) in real outdoor environment without any additional energy consumption. This work provides an impressive polymer nanocomposite with ideal full‐spectrum solar absorption and infrared emission for all‐day uninterrupted thermal energy management and conversion.

## Graphical abstract

A polymer nanocomposite with full‐spectrum strong solar/infrared absorption is prepared by large‐scale melt blending with MXene, which integrates photothermal and radiative cooling together and can be further utilized for all‐day uninterrupted electrical energy output. This polymer nanocomposite holds great promise for all‐day effective thermal energy management and conversion from the heat/cold source.

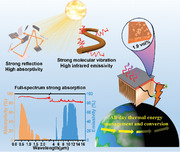

## 1 Introduction

Maintaining a comfortable temperature is a crucial aspect of modern living.^[^ ^1^, ^2^, ^3^ ^]^ The society has long had a significant demand for both heating and cooling, which account for over half of the total final energy consumption.^[^ ^4^ ^]^ The energy used for heating and cooling buildings constitutes up to 48% of the total energy consumption in buildings, making it a substantial portion of energy expenditure.^[^ ^5^, ^6^, ^7^ ^]^ Moreover, air conditioning and combustion heating contribute to various environmental issues, including atmospheric pollution and the greenhouse effect.^[^ ^8^, ^9^, ^10^ ^]^ Utilizing renewable and clean energy sources for heating and cooling, as well as exploring the conversion of heat and cold into other usable energy sources has drawn extensive attention from diverse fields for mitigating excessive energy consumption and the associated environmental problems.^[^ ^11^, ^12^, ^13^, ^14^ ^]^

Notably, the use of radiation for heat transfer holds great promise as a viable method.^[^ ^15^, ^16^, ^17^ ^]^ Radiation refers to the transport of heat in the form of both visible and non‐visible light. In particular, infrared radiation, which is invisible and has a medium wavelength, can directly dissipate heat from objects.^[^ ^18^, ^19^ ^]^ The sun, with its high temperature of ≈6000 K, constantly emits intense thermal radiation. Within this radiation, the 0.3–2.5 µm range, especially the UV‐visible light‐near‐infrared band spanning 280–2000 nm, accounts for ≈90% of the energy.^[^ ^20^ ^]^ This radiation can penetrate the Earth's atmosphere through the atmospheric window (8–13 µm), making it an ideal heat source.^[^ ^21^ ^]^ Photothermal technology harnesses solar photons to convert them into heat energy, offering advantages such as affordability, scalability. Various materials have been employed as solar energy absorbers to collect heat sources in large‐scale commercial applications.^[^ ^22^, ^23^, ^24^, ^25^, ^26^ ^]^ Additionally, the coldness present in the universe, at ≈3 K, can serve as a clean source of cold. Through radiation cooling technology, thermal radiation from ground objects is passively and continuously dissipated into the cold outer space through the atmospheric window.^[^ ^27^, ^28^, ^29^, ^30^, ^31^ ^]^ Notably, the radiation and ambient temperature is typically dynamic during daytime and nighttime. How to integrate daytime photothermal and nighttime radiative cooling in a single system is significant for efficiently harvesting energy from the sun and outer space.^[^ ^32^, ^33^, ^34^, ^35^ ^]^

The integration of photothermal and radiative cooling in a single system can not only collect green heat and cold sources for energy‐saving cooling/heating but also can be further utilized for efficient electrical output through thermoelectric devices based on the Seebeck effect.^[^ ^36^, ^37^, ^38^, ^39^ ^]^ For integrated solar heating and radiative cooling, materials with ideal full‐spectrum solar absorption and infrared emission are highly desirable for adapting the continuous dynamic variation between daytime and nighttime. Nonetheless, most current materials in nature do not have this unique spectral characteristic. For instance, metals are usually of low infrared emission and low solar absorption, ceramic and polymers are typically of high infrared emission and low solar absorption.^[^ ^40^, ^41^ ^]^ Carbon nanomaterials like graphene and carbon nanotubes, usually show high solar absorption and moderately high infrared emission, yet the absorption region is narrow and the infrared emission also cannot satisfy the requirement for radiative cooling.^[^ ^42^, ^43^ ^]^ In addition, Huang et al. recently reported the development of a polyethylene composite material containing liquid metal particles with a wide size distribution.^[^ ^26^ ^]^ The composite material demonstrated remarkable optical properties, attributed to the local surface plasmon resonance of the randomly distributed liquid metal particles. This phenomenon resulted in significant light absorption within the film, facilitated by multiple reflections and molecular vibrations. Consequently, the composite exhibited exceptional broad‐spectrum absorption characteristics spanning from 330 to 2100 nm, with absorption rates ranging from 96.9% to 99.3%. However, it is worth noting that the design of this composite material does not encompass the infrared band (8–13 µm), thereby limiting its potential for efficient thermal collection and utilization. Additionally, the complexity of processes and materials employed in most existing integrated systems to date restricts their processing flexibility. Moreover, these systems suffer from drawbacks such as environmental unfriendliness and challenges in scaling up applications.

Here, we report an integrated photothermal and radiative cooling system based on polypropylene (PP)/MXene nanocomposites fabricated by solid phase shear premixing, simply melt blending and interfacial modification. Impressively, the nanocomposites with very low MXene filler loading (1.9 vol%) exhibited high absorptivity ranging from 88.8% to 94.8% over the full‐spectrum of solar irradiation with an average value of 93.2%, as well as high emissivity ranging from 81.3% to 90.0% over the full‐spectrum mid‐infrared window with an average value of 84.2%. Real outdoor experiments revealed that the temperature of the composite can be increased by 50 °C compared to the ambient during the daytime by solar absorption, and can achieve a temperature of 8 °C lower than the ambient at night through radiative cooling via the atmospheric window. These temperature differences throughout the day can be effectively utilized for all‐day uninterrupted thermoelectric power generation by combining with a commercial thermoelectric device. The peak voltage, current, and power density of the system reached 223.5 mV, 27.9 mA, and 2.1 W m^−2^, respectively, under one standard solar irradiance (100 mW cm^−2^) during indoor simulations. Furthermore, for a real outdoor environment without any additional energy consumption, the peak voltage and current during the daytime were measured at 205.90 and 22 mA, with an output power of 4.53 mW and a power density of 1.50 W m^−2^. At night, these values remained stable at 15 mV, 1.6 mA, 24 µW, and 7.9 mW m^−2^, respectively. The all‐day uninterrupted thermoelectric generation capacity of the polymer nanocomposite performs well in most countries worldwide from the global power generation simulation. In addition, the nanocomposite exhibited enhanced mechanical strength, melt retardant and thermal conductivity properties, helping for its practical utilization in various scenarios. The work provides a feasible strategy to construct polymer nanocomposite with ideal full‐spectrum solar absorption and infrared emission for realizing highly efficient thermal energy utilization from the heat source of sun and cold source uninterruptedly.

## 2 Results and Discussion

### 2.1 Spectral Design Principle

**Figure 1:**
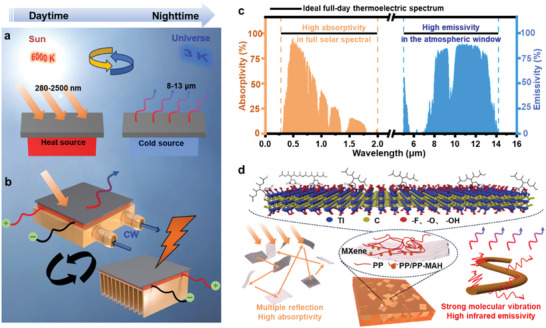
a) Schematic of solar absorption at daytime and radiative cooling at nighttime. b) Schematic of harvesting energy from the sun and outer space in one integrated system for all‐day uninterrupted electricity generation. c) Ideal spectral characteristic of materials for all‐day uninterrupted electricity generation. d) Structure schematic of MXene modified PP nanocomposite.

As illustrated in **Figure** **1**a, it is feasible to harness heat from the sun (6000 K) and cold from the universe (3 K) through radiation within the same physical region. Additionally, this concept can be combined with a commercial thermoelectric device to achieve uninterrupted power output throughout the day (Figure 1b). Figure 1c displays the spectral characteristic of the ideal material for heat and cold collection, with the shaded region representing the energy proportion of each wavelength in the total band. Specifically, to achieve effective daytime heat collection, the material should possess high absorptivity, particularly in the ultra‐high energy range of 280–2500 nm (UV‐visible‐Near‐infrared band). Conversely, to achieve cold collection at night, the material must exhibit high emissivity in the infrared band, particularly within the atmospheric transparent window to facilitate heat dissipation. As shown in Figure 1d, this study utilized MXene, which possesses high photothermal conversion efficiency, as the heat collecting material.^[^ ^44^ ^]^ The MXene was dispersed in the PP matrix to enhance absorptivity through multiple reflection of solar irradiance. Notably, the PP matrix also exhibits high infrared emissivity, thereby ensuring that the composites meet the spectral characteristic requirement for heat and cold collection from the sun and the universe, respectively.

### 2.2 Preparation, Morphology, and Optical Property of the Composites

**Figure 2:**
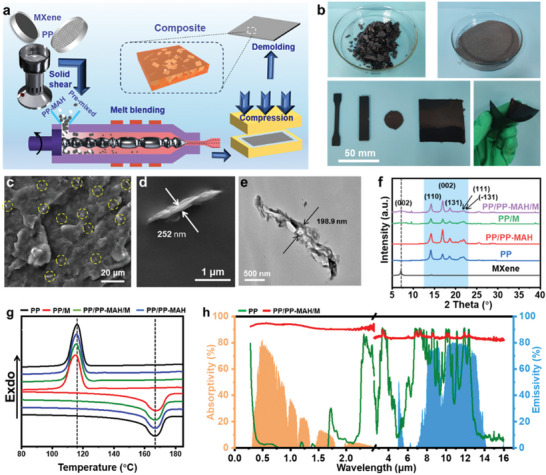
a) Schematic of preparation for PP/PP‐MAH/MXene composite. b) Digital image of MXene powder, PP/PP‐MAH/MXene composite powder, and PP/PP‐MAH/MXene composite sheets. c,d) Scanning electron microscope (SEM) images of PP/PP‐MAH/MXene composite sheet at different magnifications. e) Transmission electron microscope (TEM) image of PP/PP‐MAH/MXene composite sheet. f) X‐ray diffractometer (XRD) patterns of different composites. g) Differential scanning calorimeter (DSC) curves of different composites. h) Spectral characteristics in 0.3–16 µm of pure PP and PP/PP‐MAH/MXene composite.

In general, the non‐polar PP and polar MXene nanosheet synthesized using an in‐situ synthesis of HF etching solution (Figure S1, Supporting Information) are considered incompatible, making it challenging to achieve a homogeneous mixture, especially through direct melt blending, which often leads to agglomeration of MXene nanosheets. Impressively, by utilizing solid phase shear mixing, freeze‐dried MXene nanosheet and PP powder can be pre‐dispersed in a high‐speed agitator, facilitating the dispersion of MXene nanosheets within the PP matrix. Simultaneously, the high‐speed solid phase shear also aids in the further breakdown and dispersion of multi‐layer MXene nanosheets. As depicted in **Figure** **2**a, the pre‐mixed material was subsequently dried and subjected to melt blending in an extruder, with the addition of polypropylene grafted with maleic anhydride (PP‐MAH) compatilizer. This process further makes the MXene nanosheets disperse within the high‐flow melt through shear forces. The freeze‐dried MXene nanosheet exhibits a metallic luster and appears as relatively uniform gray‐black fine particles after pre‐mixing with PP (Figure 2b). After melt blending and molding, the composite sheet exhibits a uniform black appearance and flexibility characteristics (Figure 2b).

The PP/PP‐MAH/MXene composite exhibits a uniform dispersion of MXene nanosheets (Figure 2c) with a clear distinction between PP matrix and MXene. The transverse size of dispersed MXene ranges from 2–4 µm and its thickness measures between 200–300 nm (Figure 2d,e and Figure S2, Supporting Information), displaying minimal aggregation. Notably, compared to the MXene in the PP/MXene composite without PP‐MAH (Figures S3 and S4, Supporting Information), the size of MXene nanosheets in PP/PP‐MAH/MXene composite was significantly reduced and the dispersion was improved effectively. The decrease in MXene size, coupled with improved layer regularity, facilitates its functional integration within the composites. The X‐ray diffractometer (XRD) patterns of different composites shown in Figure 2f illustrate the characteristic diffraction peaks at 14.2°, 16.9°, 18.6°, 21.2°, and 21.9°, corresponding to the (110), (040), (130), (111), and (131) crystal faces of PP matrix, respectively. Notably, the addition of PP‐MAH introduces a discernible shift (Figure S5, Supporting Information) in the characteristic peak corresponding to the MXene crystal face (002), from 8.1° to 7.1°. This shift signifies a slightly larger space in MXene nanosheets and a more regular arrangement, indicating an improved dispersion of MXene. Additionally, according to the crystallization behavior of PP (Figure 2g and Table S1, Supporting Information), it is evident that the crystallinity itself was 51.7%. However, the random dispersion of MXene nanosheets and weak interaction between MXene and the matrix hinder the nucleation of PP, resulting in a decrease in crystallinity by 1.3% in PP/MXene composite compared with that of pure PP. Conversely, upon the addition of the compatibilizer PP‐MAH, the binding force between the matrix and MXene is significantly enhanced. As a result, MXene becomes an effective nucleating agent, promoting heterogeneous nucleation of PP and increasing the crystallinity by 0.6% in PP/PP‐MAH/MXene composite compared with that of pure PP.

Spectral characteristics in 0.3–16 µm of different composites are illustrated in Figure 2h and Figure S6 (Supporting Information). The solar absorptivity (*α*) was calculated using the formula (1):

(1) α=1−ρ−τ

where *ρ* and *τ* represent the corresponding solar reflectivity and transmission (Note S1, Figures S7 and S8, Supporting Information), respectively. PP/PP‐MAH/MXene composites show high solar absorptivity over a broadband spectrum (Figure 2h). Notably, the absorptivity in the ultra‐large band range of 280–2500 nm was measured from 88.8% to 94.8% with an average value of 93.2%. The significantly higher absorptivity compared to that of MXene film (73%, Figure S7, Supporting Information) can be attributed to the fact that a portion of the MXene film is directly reflected off after solar irradiation, whereas the scattered distribution of MXene in the as‐prepared composite facilitates multiple reflections between MXene layers. Moreover, due to the strong molecular chain vibration and side groups of the PP matrix, the composite exhibits full‐spectum high absorptivity ranging from 81.3% to 90.0% with an average value of 84.2% in the atmospheric window (Figure 2h, Note S2, Figure S9, Supporting Information). In a thermally stable state, the emissivity is equal to the absorptivity, which implies an emissivity of 84.2% in the atmospheric window.^[^ ^45^ ^]^ The exceptional absorptivity across the full‐spectrum solar band ensures that the composite can effectively harness solar energy as a heat source. Simultaneously, its high emissivity in the mid‐infrared band allows it to efficiently dissipate heat to the cold universe, thereby establishing a cold source. Notably, these resources are entirely clean and renewable in nature.

### 2.3 Indoor Simulated Thermal Energy Harvest of the Composites

**Figure 3:**
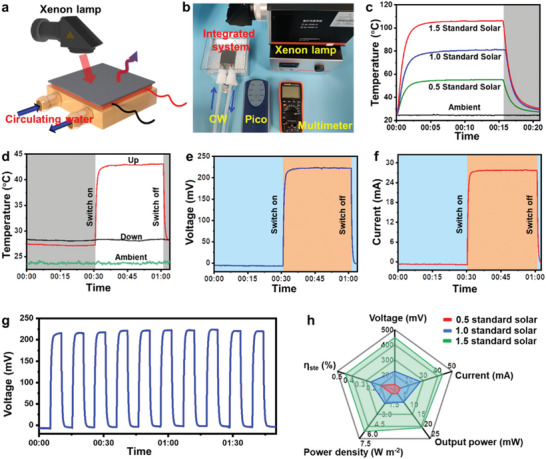
a) Schematic diagram and b) digital diagram of the indoor simulator for thermoelectric power generation. c) Real‐time temperature for indoor simulated solar heating of PP/PP‐MAH/MXene composite without circulating water. d) Real‐time temperature of simulated thermoelectricity with circulating water. e) Output voltage and (f) output current for indoor simulated thermoelectricity generation. g) Indoor simulated thermoelectricity generation for 10 cycles. h) Comparison of power generation effects of at different solar irradiance.

By integrating the heat source and cold source obtained from the system, a significant temperature difference can be easily generated. This thermal energy can be efficiently converted into electrical power using commercial thermoelectric devices. The working principle of the integrated system was succinctly demonstrated in **Figure** **3**a,b with a schematic diagram and physical representation. In essence, the integrated system continuously generates electrical energy by utilizing the temperature difference between the thermal sources collected by the composite and the circulating water. The indoor simulation of photothermal experiments shows that the integrated system can adjust the received radiation intensity by varying the power of the xenon lamp (Figure 3c). Under standard solar radiation intensities of 0.5, 1.0 (AM1.5 100 mW cm^−2^), and 1.5, the surface temperature of the PP/PP‐MAH/MXene composite could reach 55.4 °C, 81.4 °C, and 106.3 °C, respectively. As shown in Figure 3d, the temperature difference between the two sides of the integrated system remains stable at ≈1.0 °C under simulated no‐light night conditions. It should be noted that due to the mutual radiation of various objects in the room, the simulated radiative cooling effect is significantly lower than that in real outdoor conditions. Therefore, the resulting temperature difference and electrical energy are correspondingly lower than normal levels.^[^ ^46^ ^]^ Under the irradiation of simulated solar light at one standard intensity of 100 mW cm^−2^, the composite functions as the heat‐collecting component in the integrated system, leading to a rapid increase in its temperature. Conversely, the backlit side serves as the cold component. Once the integrated system reaches thermal equilibrium, the temperature at the hot end stabilizes at ≈43 °C. The observed lower temperature compared with that in Figure 3c can be attributed to the inevitable heat exchange occurring with the cold end, resulting in a stable temperature difference of ≈14.8 °C. This temperature difference generates a corresponding voltage shown in Figure 3e. Due to the relatively stable nature of the simulated solar light source, the output voltage of the integrated power generation system remains consistently stable at 223.5 mV. It is worth noting that the voltage and temperature difference exhibit synchronized variations, highlighting the sensitivity of the integrated system. Under the same conditions, the output current measures to be 27.9 mA (Figure 3f), demonstrating similar stability and sensitivity. Figure 3g shows the stability of the integrated system, with the voltage variation trend and value remaining largely unchanged during the simulated multiple switching of the xenon lamp.

Furthermore, by adjusting the simulated solar power, the electrical power generation (voltage, current, power, power density, and efficiency) of the integrated system in different environments can be significantly regulated (Figure 3h). For instance, under 0.5 standard solar irradiation, the output voltage measures to be 135.9 mV, while under 1.5 standard solar irradiation, it increases to 448 mV. The integrated system presented in this study demonstrates the capability to generate continuous electrical energy throughout the day, employing a straightforward preparation and combination process. When strategically integrated with building materials under optimal conditions, while ensuring the maintenance of thermal comfort within the room, a distinct temperature gradient can be achieved between the building's outer layer and interior, thereby enabling an efficient supply of electrical power.

### 2.4 Outdoor Thermal Energy Harvest of the Composites

**Figure 4:**
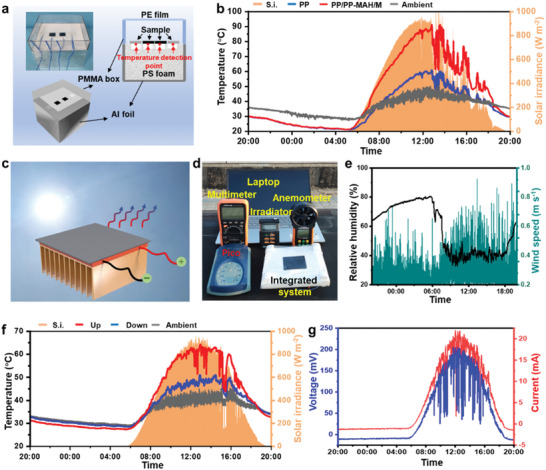
a) Schematic and digital image of outdoor heat‐ and cold‐collection device. b) Real‐time monitored solar irradiation and temperature curves for the all‐day outdoor heating and cooling experiment. c) Schematic and d) digital image of outdoor full‐day thermoelectric devices. e) Real‐time monitored wind speed and relative humidity curve during outdoor full‐day thermoelectric experiment. f) Real‐time monitored solar irradiation and temperature for the outdoor full‐day thermoelectric experiment. g) Energy output curve of the outdoor all‐day thermoelectric experiment.

The outdoor heat and cold collecting devices are illustrated in **Figure** **4**a. The sub‐environment of the unit consists of a polyester box and foam, insulated by plastic wrap and aluminum foil, demonstrating the ability of heat and cold collection by monitoring the composite and sub‐environment. Figure 4b and Figure S10 (Supporting Information) demonstrate the outdoor heat and cold collecting capacity of the integrated composite system with real‐time monitored temperature, wind speed, and relative humidity. During the night without solar irradiance, all the composites remain relatively stable temperature below the ambient of ≈8 °C. As the sun rises in the morning, the solar irradiance increases rapidly, causing the temperature of the composite to rise quickly above the ambient temperature, reaching a peak temperature of ≈90 °C. At noon under maximum solar irradiance, the temperature of PP/PP‐MAH/MXene composite was ≈50 °C higher than the ambient temperature. The exceptional heat and cold collecting capability, similar to indoor simulation, can be utilized for electrical energy output. Notably, as depicted in Figure 4c,d for an outdoor environment, the circulating water source of the integrated system can be substituted with an aluminum sink exposed to the environment to match the ambient temperature. Figure 4e displays the full‐day wind speed and relative humidity for thermoelectric application. Figure 4f illustrates that, due to the sink's influence, the lower side temperature of the integrated system remains around the ambient temperature, while the upper side temperature varies over time, primarily in line with changes in solar irradiance. At maximum irradiance and temperature, the lower ambient temperature dissipates a significant portion of the heat on the upper side, which was inevitable in an integrated system. During the sunless night, owing to the high emissivity characteristics of the composite, the upper side temperature remains relatively stable below the lower side temperature.

Figure 4g illustrates the electrical power generation effect of the integrated system throughout the entire day in a real environment. The voltage and current generated by the system exhibit a strong correlation with the temperature difference and vary with solar irradiance. During nighttime, the integrated system consistently produces stable electrical energy with values of 15 mV, 1.6 mA, and 24 µW, respectively. Throughout the day, the power output increases in accordance with the temperature difference, reaching peak values of 205.9 mV, 22 mA, and 4.5 mW, respectively. The system demonstrates effective power output without the need for additional energy input. Its remarkable passive output characteristics make it highly efficient in reducing energy consumption.

Apart from building applications, the individual integrated system also offers exceptional application flexibility and can be directly utilized for outdoor all‐day power generation. In brief, a thermal management model was built in EnergyPlus software to simulate 8760 hours of temperature change throughout the year with specific parameters (Supplementary Note S3 and Figure S11, Supporting Information). Further, the thermoelectric output capacity of the integrated system was calculated from the temperature and temperature difference. Figure S12a (Supporting Information) depicts the simulation‐based calculation of the integrated system's output power density in various countries on a global scale, which aligns closely with the experimental results. It is worth noting that the performance of the integrated system is affected by geographical region and climatic conditions, including solar radiation, humidity, cloud cover, wind speed, and ambient temperature. In particular, among the highest annual electrical power output data presented, the Faroe Islands has the lowest output power density at 0.56 W m^−2^; while Uganda has the highest output power density with a value of 2.19 W m^−2^. Six typical cities in different regions of the world were selected to fully understand the application potential of the integrated system in different climatic regions. To demonstrate the more detailed application potential of the integrated system, Figure S12b–g (Supporting Information) list the highest monthly energy output density in China (Beijing, middle latitude and low altitude), Russia (Moscow, high latitude and low altitude), the United States (Washington, middle latitude and low altitude), Australia (Canberra, middle latitude and high altitude), Brazil (Barbaria, low latitude and high altitude), and Kenya (Nairobi, low latitude and high altitude). Among them, Australia has the largest monthly variation in output power density, with values ranging from 0.32 to 1.26 (W m^−2^). While Kenya has the most stable output power density levels, varying from 0.81 to 1.20 (W m^−2^). It is evident that regions with higher irradiance receive correspondingly higher power density from the integrated system. In particular, Kenya, which is close to the equator and at a higher altitude, maintains a high level of thermoelectric output throughout the year. In a word, in most countries worldwide, this integrated system exhibits promising power output capabilities. In addition, the composites demonstrated improved mechanical properties (Figure S13, Supporting Information), flame‐resistance (Figure S14, Supporting Information) and enhanced thermal conductive performance (Figure S15, Supporting Information), helping for their applications in various scenarios in terms of all‐day uninterrupted thermal energy harvest and conversion.

## 3 Conclusion

In summary, this work reports an integrated photothermal and radiative cooling system based on PP/MXene nanocomposites fabricated by solid phase shear premixing, simpler melt blending, and interfacial modification. Impressively, the nanocomposites with very low filler loading (1.9 vol%) exhibited a high broadband absorptivity (93.2%) and a high broadband infrared emissivity (84.2%). Real outdoor experiments revealed that the temperature of the composite can be increased by 50 °C compared to the ambient temperature during the day by solar absorption, and can achieve a temperature of 8 °C lower than the ambient temperature at night through radiative cooling via the atmospheric window. This temperature difference of the nanocomposites throughout the day can be effectively utilized for all‐day uninterrupted thermoelectric power generation, yielding a peak voltage, current, and power density output of 205.90 mV, 220 mA, and 1.5 W m^−2^ during daytime without any additional energy consumption. The all‐day uninterrupted thermoelectric generation capacity of the polymer nanocomposite performed well in most countries worldwide. Moreover, the enhanced mechanical strength, flame‐retardant, and thermal conduction performance in the nanocomposites helping for its practical utilization in various scenarios, making it be of great significance to alleviate energy crisis and corresponding environmental problems.

## 4 Experimental Section

### Materials

Hydrochloric acid (98%) was sourced from the Luoyang Chemical Reagent Factory. LiF was procured from Shanghai McLean Biochemical Co., LTD. Ti_3_AlC_2_ powder (400 mesh) was supplied by Jilin 11 Technology Co., LTD. PP powder (PP‐H, GD, 150) was purchased from Dongguan Jiaqing Plastic Material Co., LTD. PP‐MAH was obtained from Jiayi Rong Polymer (Shanghai) Co., LTD. All chemicals were utilized without any pre‐treatment.

### Preparation of MXene

Ti_3_AlC_2_ was etched using an in‐situ synthesis of HF etching solution, and layered MXene was prepared using the minimum strength stratification method. To elaborate, the HF etching agent was synthesized by dispersing LiF (10 g) in a Teflon beaker containing 9 m hydrochloric acid (200 mL) and stirring for 30 min. Subsequently, Ti_3_AlC_2_ (10 g) powder was slowly added to the etching agent, and the reaction was stirred at 35 °C for 24 h. The resulting mixed slurry was then repeatedly centrifuged at 3500 rpm and washed with deionized water 3–4 times until the upper layer became colorless and transparent after stratification, while the lower layer precipitation was collected for freeze‐thaw treatment. The centrifugation process was continued until the pH reached above 6. The precipitates were collected, dispersed again with deionized water, and shaken by hand for 10 minutes before freeze‐drying.

### Preparation of PP/PP‐MAH/MXene Composites

PP powder and freeze‐dried MXene powder were coalesced in a high‐speed mixer for solid‐phase shear premixing, followed by the addition of PP‐MAH. The premixing was divided into several times at room temperature to reduce the degree of oxidation. Subsequently, the composite masterbatch was obtained through drying and extrusion blending, temperature of each region was 170, 180, 190, 180, 170 °C. The masterbatch was then granulated and molded into various forms for subsequent characterization. The molding temperature was 180 °C, and the whole process included 2 min‐prepressure under 5 MPa and 4 min‐supercharging under 10 MPa. Similar procedures were employed to prepare PP, PP/MXene, and PP/PP‐MAH composites.

### Characterization

The morphology of the prepared MXene and the cross–section of the composite were characterized using a ZEISS GeminiSEMM 300 SEM operating at 15 KV. The dimensional state of the MXene in the matrix was examined using a Japan JEOL JEM‐2100Plus TEM operating at 20 KV. The thermal properties of the material were analyzed using a DSC 200 F3, with a heating rate of 10 °C min^−1^ from 30 °C to 190 °C under a nitrogen atmosphere. The crystal information of the material was obtained using a Bruker D8 Advance XRD with a scanning speed of 5° min^−1^ in the range of 5° to 90°. Infrared reflectance (*ρ*) and transmittance (*τ*) were measured using an FTIR spectrometer (Spotlight 200i, PerkinElmer) equipped with an infrared integrating sphere. The reflectance (*ρ*) and transmittance (*τ*) of the solar spectral band were measured using a UV‐VIS‐NIR spectrometer (Lambda 1050+, PerkinElmer) with an integrating sphere attachment. The infrared emissivity (*ε*) and absorptivity (*α*) were calculated using the formula in Notes S1 and S2 (Supporting Information), and the weighted emissivity and absorptivity for specific bands were determined. Mechanical properties testing was performed using an electronic universal testing machine (UTM6104) at a constant rate of 5 mm min^−1^ by cutting the sheets into dumbbell‐shaped specimens (4 × 25 mm^2^). To evaluate flame retardancy and drip resistance, a circular sheet with a diameter of 25 mm and a thickness of 1 mm was employed for testing. After the composites were ignited, the alcohol lamp was removed, and the properties were judged by observing the droplet phenomenon in the combustion process and recording the time required for burnout. The prepared composite material (25 mm in diameter and 1 mm in thickness) was placed on a hot table (IKA C‐MAG HP4) at 100 °C, and the change of its surface temperature over time was recorded with an infrared camera (FILR E75).

### Indoor Simulations With Xenon Lamp

The integrated system employed a xenon lamp, equipped with filters, to accurately replicate real solar irradiance. The composite material (55 mm in length and width, 0.3 mm in thick) and the aluminum plate, which were connected to circulating water at room temperature, were positioned on opposite sides of the thermoelectric device (TGM‐336–1.4–1.5, Shenzhen Futian district new spring electronic firm). The composite material was placed on the top, followed by the generating sheet in the middle, and the aluminum plate at the bottom. The gap between the three components was filled with thermal conductive silicone grease. To minimize the influence of external heat convection and conduction, the power generation body of the integrated system was enclosed in a non‐top acrylic box, pre‐filled with foam, and covered with PE plastic wrap. Furthermore, the acrylic box was wrapped with aluminum foil to minimize unnecessary light absorption. During daytime, a Xenon lamp source (CEL‐PE300L‐3A) with a filter (AM 1.5) was utilized to simulate solar irradiation on the integrated system. The irradiation intensity was monitored beforehand, and the lamp was removed to replicate a non‐solar irradiation environment during nighttime. Temperature measurements were performed using a K‐type thermocouple (DT1310 LIUHUAJIN) with PICO (TC‐08), while voltage and current readings of the integrated system were recorded using a digital multimeter (VICTOR 86E). The indoor relative humidity was ≈72%.

### Outdoor Application

The outdoor experiments were conducted on the rooftop of Zhengzhou University, China (east longitude: 113°32′; northern latitude: 34°48′, August 3–4, 2023). For the heat and cold collection test, a composite sheet of varying proportions was affixed to the foam and placed inside a transparent acrylic box. The top of the box was covered with a layer of PE plastic wrap to minimize the impact of external heat convection and conduction. To reduce unnecessary light absorption, the acrylic box was wrapped with aluminum foil. Temperature measurements of the cavity under the sheet were recorded using K‐type thermocouples and PICO. Real‐time monitoring of solar irradiance, wind speed, ambient temperature, and relative humidity at the test site was conducted using a solar power meter (TES1333R) and a digital anemometer (PM6252B, PEAKMETER). In the outdoor thermoelectric application, the test device was similar to the indoor setup, except for the replacement of the circulating water plate with an aluminum sink. The output electrical energy was detected and recorded using a digital multimeter (V86E).

## Conflict of Interest

The authors declare no conflict of interest.

## Supporting information

Supporting Information

## Data Availability

The data that support the findings of this study are available from the corresponding author upon reasonable request.
