# Supplementary material for: A Polymer Nanocomposite with Strong Full‐Spectrum Solar Absorption and Infrared Emission for All‐Day Thermal Energy Management and Conversion
Source: Adv Sci (Weinh). 2024 Feb 11;11(15):2308200. doi: 10.1002/advs.202308200 (PMC11022738; doi:10.1002/advs.202308200)
Supplement: Supplementary file 1 — Supporting Information [file ADVS-11-2308200-s001.pdf]

ADVANCED SCIENCE Open Access

Supporting Information

for *Adv. Sci.*, DOI 10.1002/advs.202308200

A Polymer Nanocomposite with Strong Full-Spectrum Solar Absorption and Infrared Emission for All-Day Thermal Energy Management and Conversion

*Xiangxin Li, Zipeng Zhang, Xueting Zhang, Yanxia Cao, Yanyu Yang, Wanjie Wang and Jianfeng Wang**

Supporting Information

A polymer nanocomposite with strong full-spectrum solar absorption and infrared emission for all-day thermal energy management and conversion

*Xiangxin Li, Zipeng, Zhang, Xueting Zhang, Yanxia Cao, Yanyu Yang, Wanjie Wang, Jianfeng Wang**

School of Materials Science and Engineering, Zhengzhou University, Zhengzhou 450001, China

*Corresponding Author: jfw@zzu.edu.cn

Supplementary Note S1: Calculation of average solar spectral absorptivity

The average spectral absorptivity at 280-2500 nm of different composites is the irradiance-weighted average of its spectral absorptivity and was calculated according to the follow formula (1):

$$\alpha = \frac{\int_{280}^{2500} \alpha(\lambda) i(\lambda)\, d\lambda}{\int_{280}^{2500} i(\lambda)\, d\lambda} \quad (1)$$

where *α* (*λ*) is spectral absorptivity obtained on the relation, *ie.* $\alpha = 1 - r - t$ (*α*, *r* and *t* are spectral absorptivity, reflectivity, and transmission, respectively). The spectral reflectivity (*r*) and transmissivity (*t*) were obtained using UV-VIS-NIR spectrometer (Lambda 1050+, PerkinELmer) accompanied with an infrared integrating sphere. *i* (*λ*) is the solar spectral irradiance (W m^{-2} nm^{-1}) obtained from ASTM standard G173-03.

Supplementary Note S2: Calculation of average mid-IR spectral emissivity

The average mid-IR spectral emissivity at 8-13 μm of different composites was calculated according to the following equations (2):

$$\varepsilon = \frac{\int_{8}^{13} \varepsilon(\lambda) E(\lambda)\, d\lambda}{\int_{8}^{13} E(\lambda)\, d\lambda}$$

(2)

where *E (λ)* and *ε (λ)* represent spectral radiance emitted from blackbody at *T* (300 K) and the *ε* at wavelength *λ* measured at room temperature (298 K), respectively.

Supplementary Note S3: Calculation of thermoelectric simulation

The entire thermoelectric simulation is conducted using EnergyPlus as the underlying framework. Initially, a model resembling the outdoor scenario is constructed, wherein the thermoelectric sheet is layered with a composite material on its upper side, while the lower side is subjected to the ambient temperature. EnergyPlus requires several main input

parameters, namely: geometry model, weather conditions and internal load. Geometric models include structures and materials. In an energy simulation model, Spaces (such as hot and cold ends) are defined by spatial boundaries. The second input is weather data, including climate parameters such as humidity, wind speed, and outside temperature. Finally, the internal load includes various simulation parameters.

Briefly, a ceramic material is constructed in a space of suitable size as the boundary of the thermoelectric sheet (Figure S10). The upper surface of the thermoelectric sheet is closely bonded to the composite material, and the lower surface is exposed to the environment. The effective area of thermoelectric sheet and composite material is 55×55 mm^2. The weather data is derived from the 2022 annual weather data file, and the specific locations are selected from the capitals of each country. The thermoelectric sheet (TGM-336-1.4-1.5) contains 241 pairs of semiconductor thermoelectric legs with an average resistance of 2.5 Ω. The other parameters were compared with characterization.

First, the total number of pairs (N_T) of thermoelectric wafer semiconductor modules can be calculated by formula (3):

$$N_T = N_S \times N_P \quad (3)$$

where N_S is the number of pairs of series semiconductor modules and N_P is the logarithm of parallel semiconductor modules.

The simulated output power can be calculated by formula (4):

$$P = V \times I = \frac{N_T \times (S_M \times T_D)^2}{4 \times R_M} \quad (4)$$

where P, V and I are power, voltage and current respectively; N_T is the total number of semiconductor module pairs calculated in formula (3); S_M is the average Seebeck coefficient

of the semiconductor module; R_M is the average impedance of the semiconductor module; T_D is the temperature difference used in the simulation.

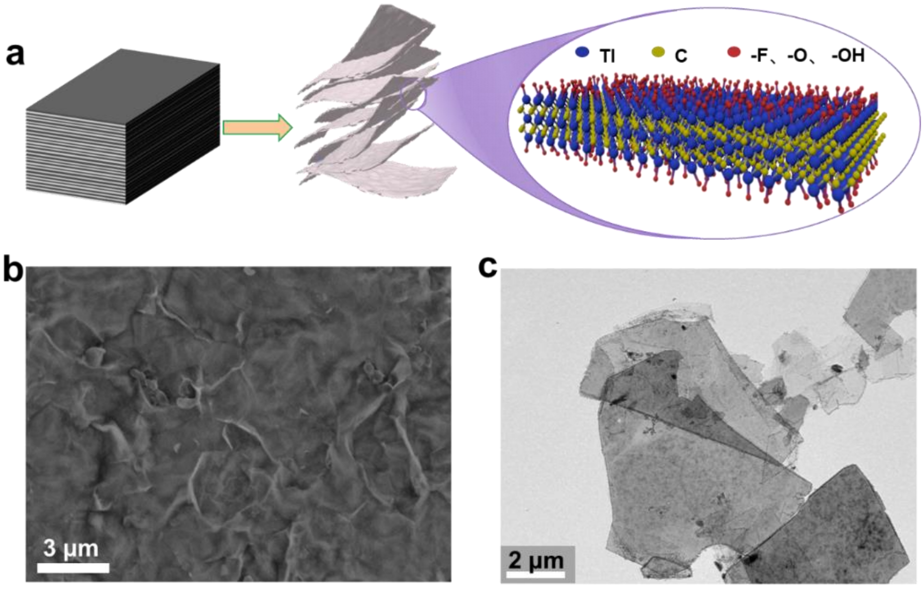

Figure S1. (a) Schematic diagram of synthesis of MXene nanosheets; (b) SEM image and (c) TEM image of MXene nanosheets.

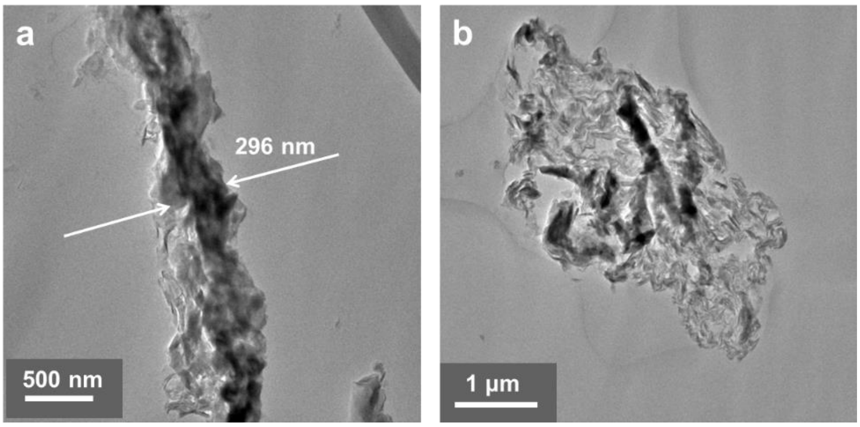

Figure S2. TEM images of PP/PP-MAH/MXene composite.

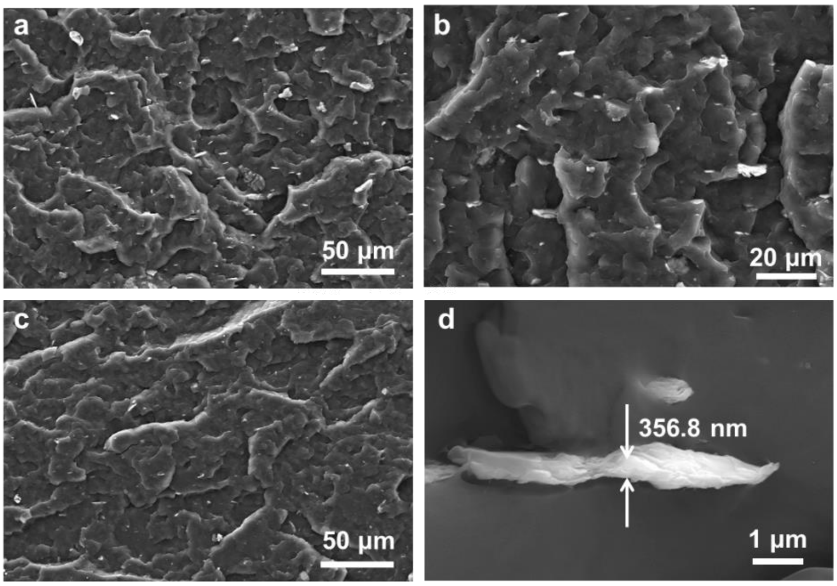

Figure S3. SEM images of PP/MXene composite at different magnifications.

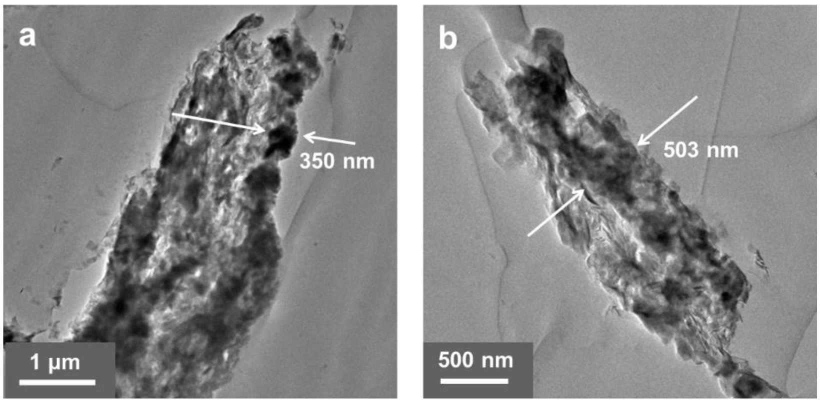

Figure S4. TEM images of PP/MXene composite at different magnifications.

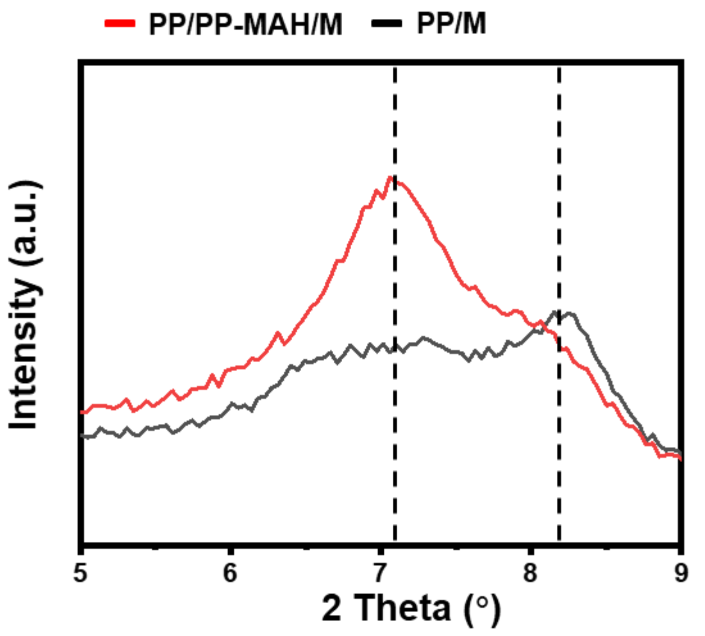

Figure S5. XRD patterns of PP/MXene and PP/PP-MAH/Mxene composites.

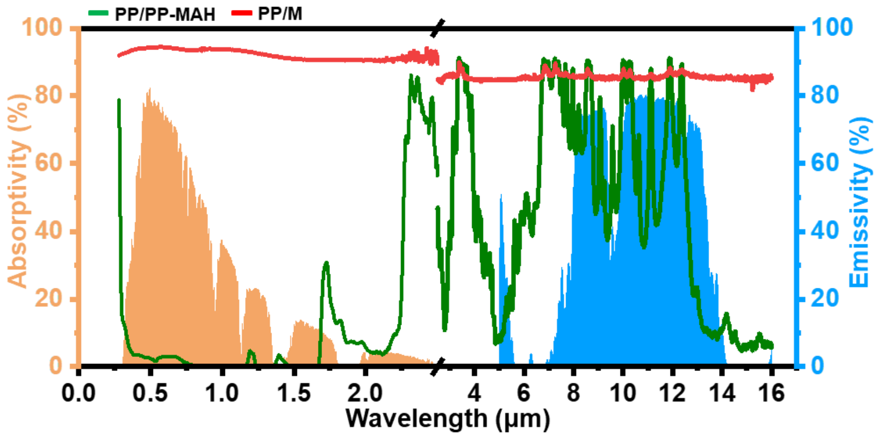

Figure S6. Spectral characteristics in 0.3-16 μm of PP/MXene and PP/PP-MAH.

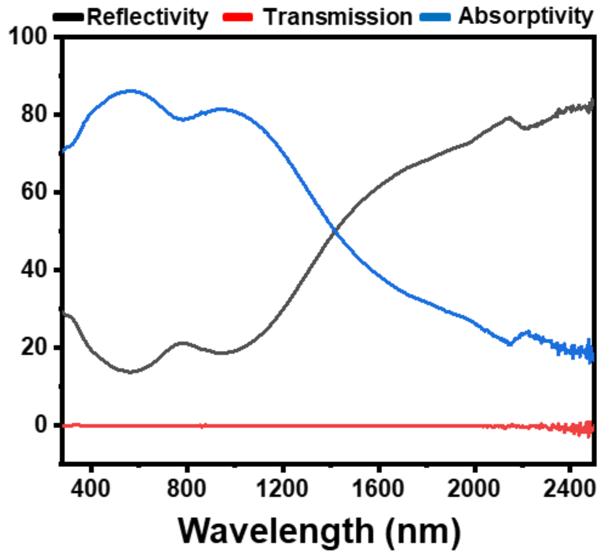

Figure S7. UV-visible-NIR reflectivity, transmission, and absorptivity of MXene.

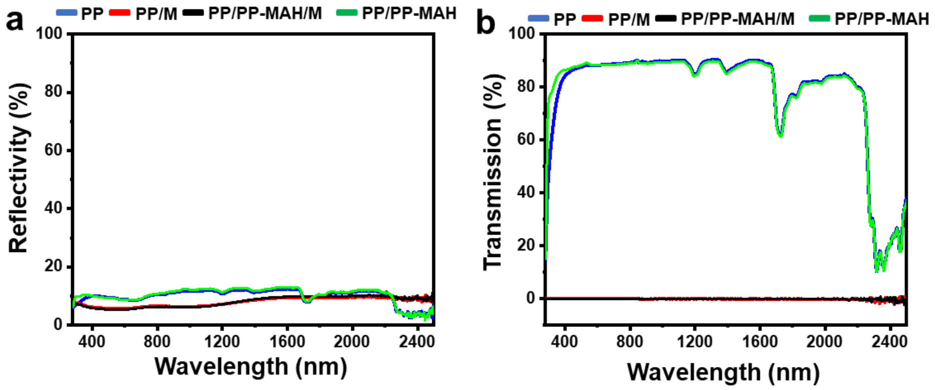

Figure S8. (a) UV-visible-NIR reflectivity of different composites; (b) UV-visible-NIR transmission of different composites.

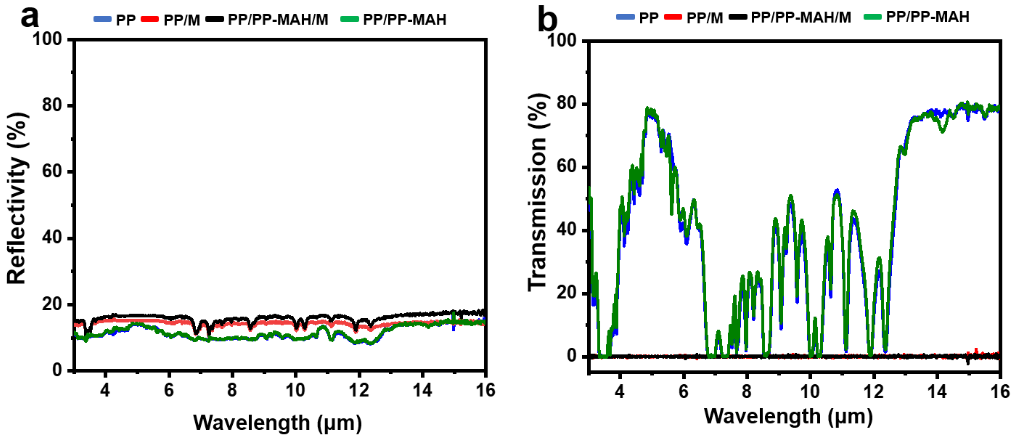

Figure S9. (a) Infrared reflectivity spectrum and (b) infrared transmission spectrum of different composites.

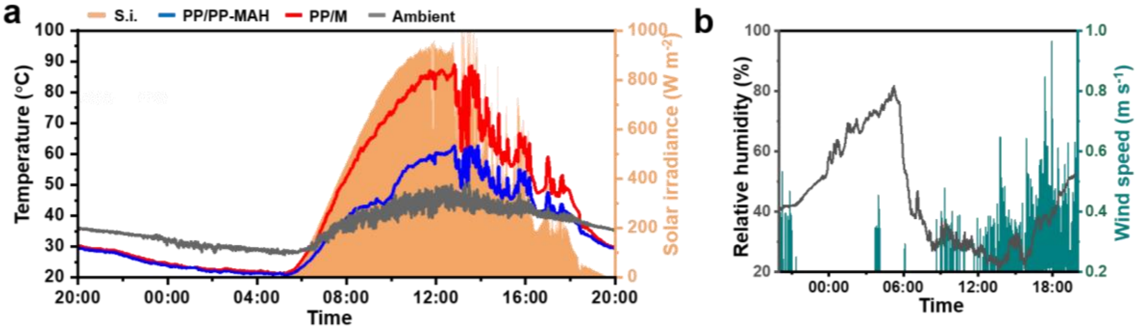

Figure S10. (a) Temperature of PP/MXene and PP/PP-MAH; (b)wind speed and relative humidity during thermal energy harvest.

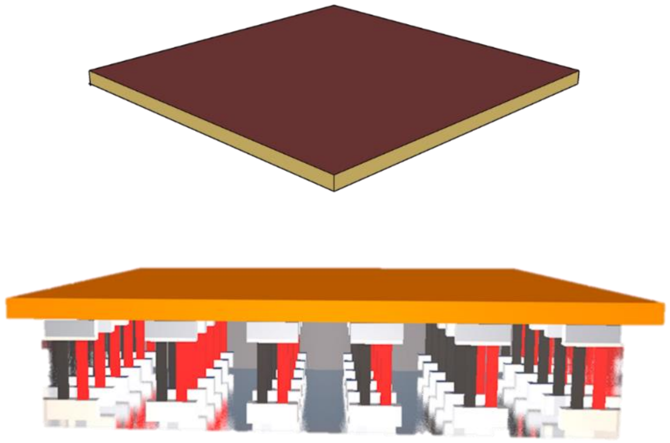

Figure S11. Model for EnergyPlus simulation.

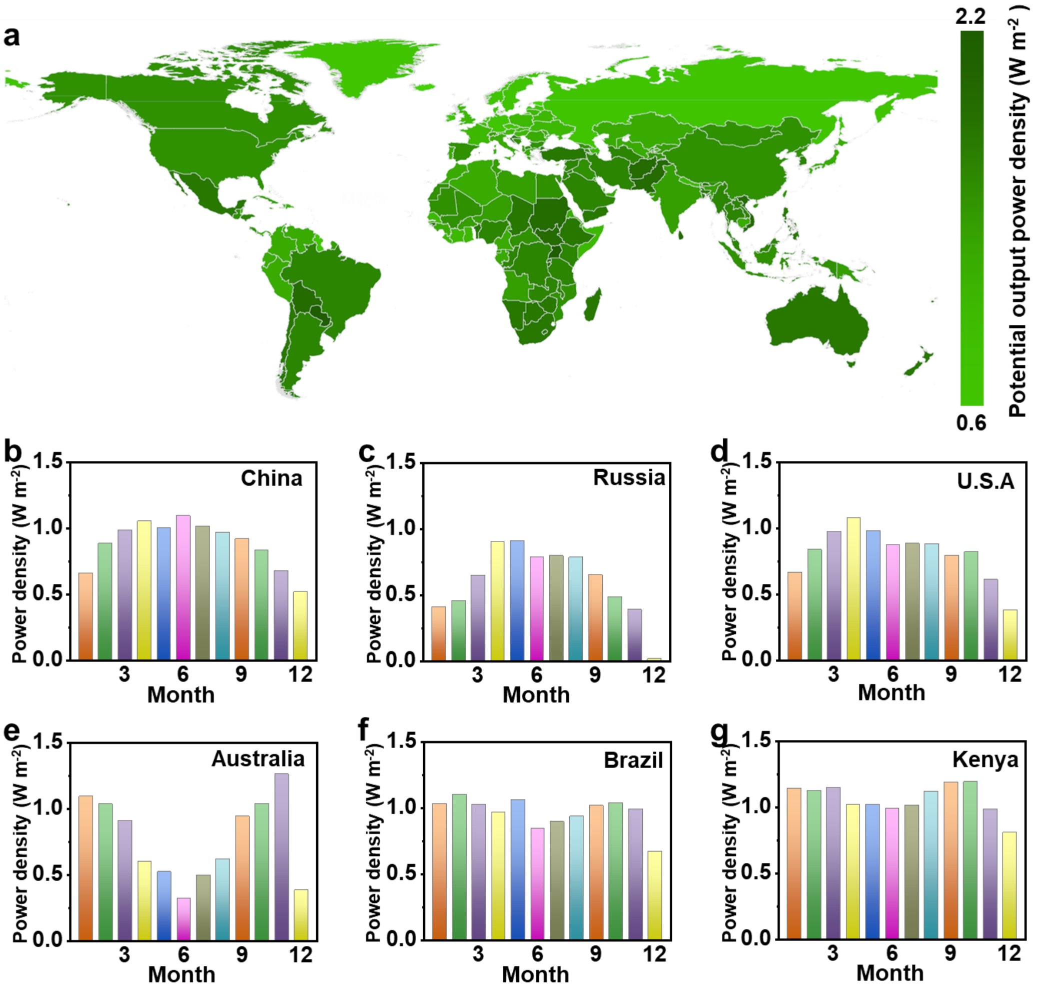

Figure S12. a) Global power generation simulation of different countries. b-g) The monthly energy output of typical countries around the world.

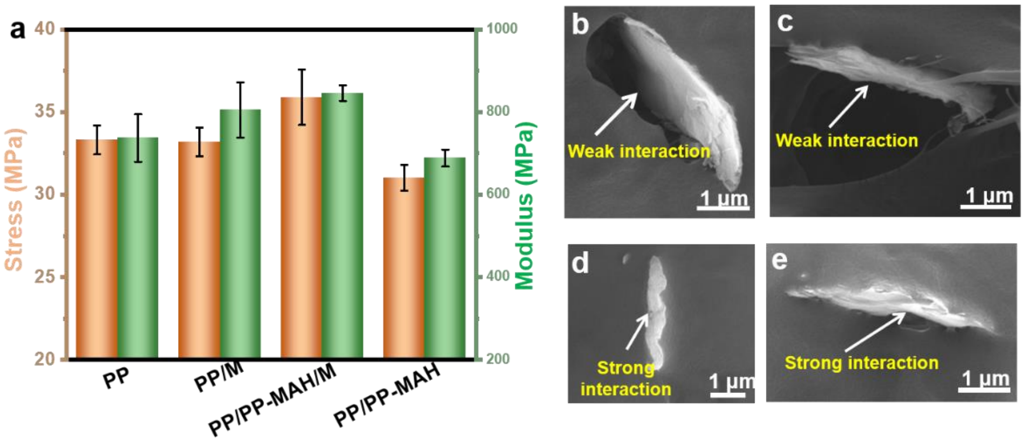

Figure S13. (a) Mechanical property of different composites. Cross-section SEM images of different composite after tensile fracture: (b,c) PP/MXene composite; (d,e) PP/PP-MAH/MXene composite. Figure S13a presents the mechanical properties of the composites, wherein the tensile strength and Young's modulus of the PP/PP-MAH/MXene composite exhibit significant improvements compared to the other components. Specifically, the tensile strength and Young's modulus were measured to be 35.9 MPa and 846.1 MPa, respectively, which were 15.9% and 22.9% higher than those of PP matrix. Figure S13b-S13e showcases the cross-sectional SEM images of the composites after undergoing tensile testing. In the PP/MXene composites, the presence of MXene was predominantly observed within the voids generated by material destruction, indicating a weak interaction between MXene and the matrix (Figure S13b and S13c) and making MXene tend to be pulled out under tensile stress. The addition of a compatibilizer enhances the binding force between the MXene nanosheet and the matrix, thereby enabling MXene to act as a strengthening phase within the matrix (Figure S13d and S13e).

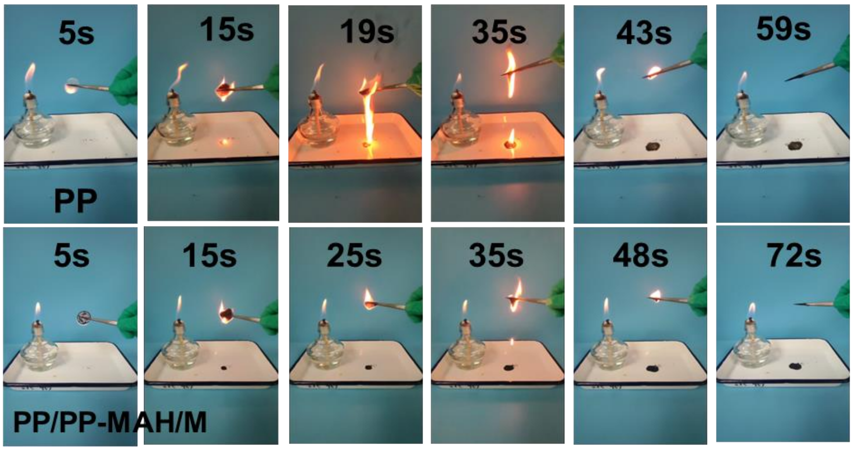

Figure S14. Melt droplet resistance property of different composites. Figure S14 illustrates the droplet resistance of different composites towards flame. Typically, the pure PP sheet exhibits rapid burning with continuous droplets, while the droplets continue to burn. In contrast, the incorporation of MXene introduces an inorganic MXene layer that functions as a flame retardant and provides support within the sheet, resulting in prolonged combustion time. Moreover, the droplet phenomenon was significantly mitigated, and the dripping melt no longer sustains combustion. Conversely, the addition of the compatibilizer does not exert a significant influence on the combustion behavior, indicating that the anti-dripping effect was primarily attributed to the presence of MXene.

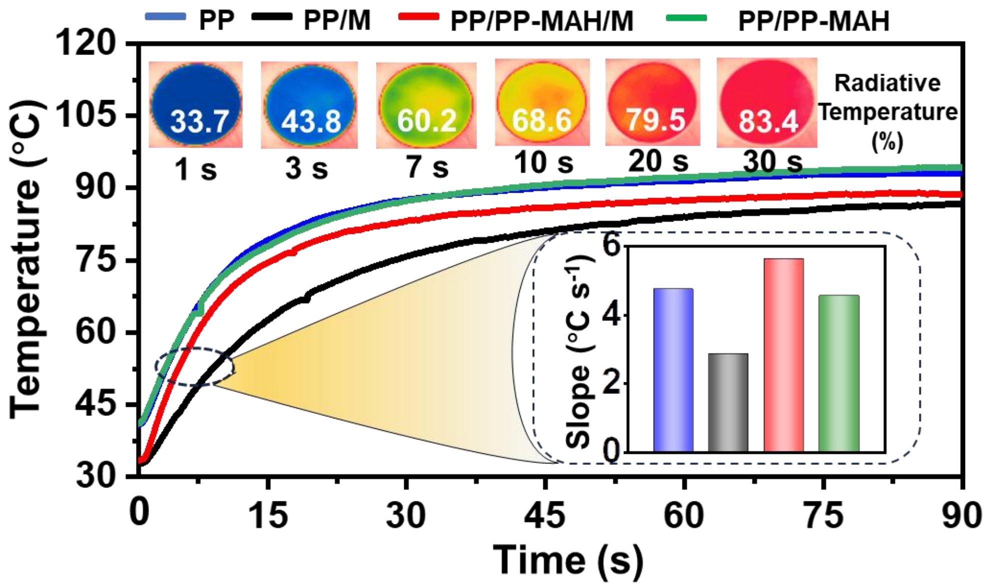

Figure S15. Heat-conducting droplet resistance property of different composites (the infrared images in the illustration are PP/PP-MAH/MXene). The PP/PP-MAH/MXene composite exhibits enhanced thermal conduction property. Under the influence of a hot table set at a temperature of 100 °C, the composites achieved thermal equilibrium after approximately 3 minutes. The accompanying illustration depicts the heating rate and corresponding infrared thermal images, clearly indicating that PP/PP-MAH/MXene exhibits superior thermal conductivity. These findings serve as evidence that the composite can acquire a range of properties without necessitating intricate procedures, thereby broadening its potential applications.

Table S1. Detail information of DSC results of different composites.

	PP	PP/MXene	PP/PP-MAH/MXene	PP/PP-MAH
Crystallinity [%]	51.7	50.4	52.3	49.8
Crystal peak area [J g^{-1}]	108.0	100.3	104.0	104.1
Crystal peak temperature [°C]	116.1	115.1	115.5	115.8
Crystal peak width [°C]	8.6	9.9	9.3	8.9
Melting peak temperature [°C]	166.8	167.3	166.9	166.9
Melting peak width [°C]	15.4	17.0	16.1	15.0
